# Multidisciplinary approach to community engagement in global public health research

**DOI:** 10.12688/wellcomeopenres.19974.1

**Published:** 2023-10-06

**Authors:** Oluwafemi Adeagbo, Mbuzeleni Hlongwa, Malak Tleis, Priyanka Dubey, Rima Afifi, Azeez Butali

**Affiliations:** 1 Department of Community and Behavioral Health, The University of Iowa, Iowa City, Iowa, 52242, USA; 2 Department of Sociology, University of Johannesburg, Auckland Park, Gauteng, 2006, South Africa; 3 Public Health, Societies and Belonging, Human Sciences Research Council, Pretoria, Gauteng, 0083, South Africa; 4 School of Nursing and Public Health Medicine, University of KwaZulu-Natal, Durban, KwaZulu-Natal, South Africa; 5 Department of Epidemiology and Population Health, American University of Beirut, Beirut, Beirut Governorate, Lebanon; 6 Department of Oral Pathology, Radiology and Medicine, The University of Iowa, Iowa City, Iowa, 52242, USA; 7 Iowa Institute for Oral Health Research, The University of Iowa, Iowa City, Iowa, 52241, USA

**Keywords:** Community engagement, research integrity, ethics, global health, Africa

## Abstract

Recently, there has been a renewed interest in the role of community engagement in knowledge production and ethical issues such as ‘helicopter research’, indicating exploitative research activities of some researchers as well as short-term relationships with research communities especially in low- and middle-income countries. This approach is detrimental to both communities and the larger scientific community as this may breed mistrust. Major institutions such as the National Institute of Health and Care Research in the United Kingdom have highlighted the importance of community engagement as a tool to improve the reach, quality, and impact of the research by incorporating the voices and concerns of marginalized communities. Similarly, in its 2022 guidance, the American Society for Human Genetics (ASGH) highlights the need to address underrepresentation in genomics research through community engagement. Establishing ethical and meaningful long-term relationships can be challenging especially for researchers who are not members of the community or those from other countries. This article describes how ‘community-engaged research’ can address some ethical challenges in global public health in different cultural settings.

**Disclaimer:** The views expressed in this article are those of the author(s). Publication in Wellcome Open Research does not imply endorsement by Wellcome.

Community engagement (CE), defined as a participatory approach that involves communities in decision-making, planning, design, governance, and implementation of programs and interventions ^1^ has been studied and utilised extensively at multiple levels of health research ^1– 3^. Many institutions, including the National Institute of Health and Care Research (NIHR) in the United Kingdom have highlighted the importance of CE as a tool to improve the reach, quality, and impact of the research by incorporating the voices and concerns of marginalized communities ^4^. The concept of community engagement builds on existing ethical guidelines and frameworks for ethical research and participatory action research techniques, resulting in some variability in how it is defined, developed, and implemented in global public health ^5^. The growing recognition of ethical considerations in research has strengthened the case for many funding institutions, international research ethics guidelines, and research institutions to begin advocating and oftentimes mandating community engagement incorporation in new studies ^4^. As research progresses to reflect greater attention to ethical frameworks and the ways in which communities can be harmed or exploited in this work, CE has been a critical tool in providing insight and frameworks to navigate these challenges effectively ^2^.

Recently, there has been a renewed interest in the pivotal role of community engagement in knowledge production and addressing ethical issues such as the problematic phenomenon known as ‘helicopter research’. This term refers to exploitative research activities conducted by some researchers as well as short-term relationships with research communities especially in low- and middle-income countries (LMICs) ^6^. Such an approach is not only detrimental to communities involved but also erodes trust within the larger scientific community. In order to rectify these concerns, many scientific institutions have advocated for a shift towards more community-engaged research (CEnR) to address underrepresentation, health disparities, research injustices, and to promote ethical partnerships and recognise collective expertise of all stakeholders throughout the research cycle ^7^. For example, in its 2022 guidance, the American Society for Human Genetics (ASGH) highlighted the need to address underrepresentation in genomics research through community engagement ^7^. However, establishing ethical and meaningful long-term relationships can be challenging, particularly for researchers who are not members of the community or those from other countries.

At the just concluded Oxford Global Health and Bioethics International Conference (June 26–27, 2023) organized by the Ethox Centre/Wellcome Centre for Ethics and Humanities at the University of Oxford and John Hopkins Berman Institute for Bioethics, a group lightning talk (panel session) entitled “ *Community Engagement and Ethical Issues in Global Health Research”* was organized. This panel session was well attended both physically and virtually signifying the importance of CEnR among global health researchers and funders. Five speakers in the session presented CEnR from different research settings and perspectives. Facilitators and participants stimulated discussions on how ‘community-engaged research’ can address some ethical challenges in global public health, e.g., ‘helicopter’ or ‘colonial’ research, underrepresentation in genetics and genomics research, research in humanitarian settings etc. In their intervention study among young Syrian refugees living in Lebanon, Tleis *et al.* described how the formation of Community Alliance Committee (CAC) reduces power inequities and acknowledging capacity, knowledge, and expertise within community. The CAC members questioned the original plans for recruitment, provided feedback and edits to all data collection instruments, interrogated the reasoning behind intervention processes, and provided important contextual cues that guided decision making around the collection of hair to measure cortisol in this religious community. They also highlighted how the political landscape in Lebanon generated some concerns around legality/illegality of Syrian refuges/asylum seekers that eventually affected the participation of some participants in their research. Similarly, Dubey and Afifi emphasized the importance (including challenges) of ‘ *trust*’ building with community-based organisations) in their study that explored the lived experiences of menstrual hygiene management among transgender and non-binary people in urban India. They highlighted their challenges especially the push-back they received from the community. Due to the initial decline from community members, the authors reflected on their positionality and how it affected their initial relationships with the community. Through reflexivity and positionality, *trust* was a key element in facilitating ethical and effective research in their project.

In their study of the impact of secondary findings (SF) among orofacial cleft (OFC) families in Africa, Butali and Oladayo investigated participants consent, concerns, and opportunities to access novel aspects of SF in their OFC cohort and, the role of providers and community in improving genomic education, knowledge, and utilization using whole genome sequencing. Their presentation focused on global ethical debate in genomic research and lessons learned from their cleft cohort, providers, and community gatekeepers. Applying the *utilitarian principle* ^8^ (greater good for the majority), Adeagbo explored community workers concerns about a home-based HIV testing trial that they implemented in poor communities in rural South Africa. Although HIV testing uptake increased by 55% among men (target population) due to the financial incentives (R50[$3] food vouchers) offered, some of the implementers (who were also community members) faced serious resentment (including verbal abuse) from participants and community members because of the financial incentive) they distributed during the study. Adeagbo raised some ethical questions about ‘greater good’ in this context. Is it ‘morally right’ to ignore the safety of the implementers in this context since the financial incentive has achieved the ‘greater good for the target population’ in the community? How do we (global health researchers) ensure the safety and wellbeing of our local partners in a situation like this?

Finally, presenting data from multiple community-based HIV projects and existing literature reviews, Hlongwa and Adeagbo shed light on the scarcity of community-engaged research on pre-exposure prophylaxis (PrEP) among adolescent boys and young men (ABYM) in South Africa. Despite South Africa’s roll-out of PrEP in 2016 and growing research on this topic, there remains a concerning dearth of community-engaged research focused on PrEP and post-exposure prophylaxis among ABYM compared to similar research among adolescent girls and young women. They teased out some of the ethical challenges of one-sided research and how closing this knowledge gap is critical to addressing an important blind spot in the response to HIV. They argued that addressing this knowledge gap is critical because it hinders efforts to combat HIV/AIDS as a public health threat. Thus, to accelerate the pace towards ending HIV/AIDS as a public health threat, they argued that it is crucial to tackle the underlying reasons for the current disparities in HIV research efforts and ensure a more inclusive and equitable approach to understanding and preventing HIV transmission among ABYM.

In conclusion, the different studies presented in the panel session highlighted the need to magnify underrepresented or marginalized population voices through CEnR. It also flipped the narratives about who the ‘expert’ is in global health research. Finally, it is safe to argue that mutual respect (including cultural humility) and benefits are key elements of CEnR to co-produce knowledge with the communities in global public health research.

## Data Availability

No data are associated with this article.
