# Supplementary material for: Multidisciplinary approach to community engagement in global public health research
Source: Wellcome Open Res. 2023 Nov 27;8:429. Originally published 2023 Oct 6. [Version 2] doi: 10.12688/wellcomeopenres.19974.2 (PMC10714101; doi:10.12688/wellcomeopenres.19974.2)
Supplement: Peer review history

# Reviewer report: Reviewer response for version 2

**DOI:** 10.21956/wellcomeopenres.22788.r70569

**Published:** 2023-12-11

**Authors:** Brenda Yankam

**Affiliations:** 1 University of Nigeria, Nsukka, Enugu, Nigeria

All comments have been well addressed.

 Hence, the manuscript should be accepted as it is.

Does the article adequately reference differing views and opinions?

Yes

Are all factual statements correct, and are statements and arguments made adequately supported by citations?

Yes

Is the Open Letter written in accessible language?

Yes

Where applicable, are recommendations and next steps explained clearly for others to follow?

Partly

Is the rationale for the Open Letter provided in sufficient detail?

Yes

Reviewer Expertise:

Community engagement, infectious diseases, HIV/AIDS, Quantitative and qualitative data analysis

I confirm that I have read this submission and believe that I have an appropriate level of expertise to confirm that it is of an acceptable scientific standard.

# Reviewer report: Reviewer response for version 2

**DOI:** 10.21956/wellcomeopenres.22788.r70568

**Published:** 2023-12-11

**Authors:** Caroline Agboola

**Affiliations:** 1 Jindal Institute of Behavioral Sciences, Sonipat, India

The revised article is okay.

Does the article adequately reference differing views and opinions?

Yes

Are all factual statements correct, and are statements and arguments made adequately supported by citations?

Yes

Is the Open Letter written in accessible language?

Yes

Where applicable, are recommendations and next steps explained clearly for others to follow?

Yes

Is the rationale for the Open Letter provided in sufficient detail?

Partly

Reviewer Expertise:

NA

I confirm that I have read this submission and believe that I have an appropriate level of expertise to confirm that it is of an acceptable scientific standard.

# Reviewer report: Reviewer response for version 1

**DOI:** 10.21956/wellcomeopenres.22119.r68253

**Published:** 2023-11-09

**Authors:** Caroline Agboola

**Affiliations:** 1 Jindal Institute of Behavioral Sciences, Sonipat, India

Thank you for the opportunity to review the open letter, Multidisciplinary approach to community engagement in global public health research.

 The document provides important insights into community engagement research and the ethical issues involved therein, particularly through a multidisciplinary lens. Examples of community engagement research in Lebanon, India, South Africa and other parts of Africa were discussed.

 The document is good but can be better. My suggestions on how to improve the quality of the document is as follows:

1. While the objective of the writing was clearly laid out in the abstract, this was not followed through in the body of the writing. Objective(s) of the writing should be included within the body of the writing.
2. A clear link should be established between the panel session that was mentioned in the writing and the writing. The answer to this question "Why the specific panel session and not any other?" will help to shed more light on the missing link. In other words, the importance of the panel session to the writing must be clearly demonstrated.
3. Similarly, the relationship, if any, between the five panelists and the subsequent studies that were cited should be discussed.
4. The names of the five speakers of the panel session should be mentioned in order to give more depth of meaning to the writing.

Does the article adequately reference differing views and opinions?

Yes

Are all factual statements correct, and are statements and arguments made adequately supported by citations?

Yes

Is the Open Letter written in accessible language?

Yes

Where applicable, are recommendations and next steps explained clearly for others to follow?

Yes

Is the rationale for the Open Letter provided in sufficient detail?

Partly

Reviewer Expertise:

Sociological research, Criminology and Qualitative research.

I confirm that I have read this submission and believe that I have an appropriate level of expertise to confirm that it is of an acceptable scientific standard, however I have significant reservations, as outlined above.

# Response

**Published:** 2023-11-23

**Authors:** Oluwafemi Adeagbo

**Affiliations:** Community and Behavioral Health, The University of Iowa, Iowa City, Iowa, USA

Dear Reviewer, Thank you very much for your insightful comments. We have revised the manuscript as suggested. 

 Thank you

# Reviewer report: Reviewer response for version 1

**DOI:** 10.21956/wellcomeopenres.22119.r68251

**Published:** 2023-10-19

**Authors:** Brenda Yankam

**Affiliations:** 1 University of Nigeria, Nsukka, Enugu, Nigeria

The open letter titled "Multidisciplinary approach to community engagement in global public health research" provides valuable insights into the importance of community engagement in research and highlights key ethical challenges.

 The study addresses a highly relevant and timely topic in the field of global public health. It effectively discusses the ethical considerations related to community engagement, 'helicopter research,' and underrepresentation in research.

 The inclusion of case studies from the Oxford Global Health and Bioethics International Conference adds practical depth to the discussion. These case studies illustrate the real-world application of community engagement principles, making the content more relatable and insightful.

 The study adopts an interdisciplinary perspective by considering issues in genomics, HIV research, and other areas of public health. This multidisciplinary approach adds breadth to the discussion and showcases the universal relevance of community engagement.

 The study does a commendable job of addressing ethical considerations in research, including the potential harm to communities, power imbalances, and the need for mutual respect and cultural humility. These discussions are crucial for ensuring ethical and responsible research practices.

 My recommendations are as follows

1. While the study provides valuable information, there is room for improvement in terms of clarity and structure. The letter could benefit from a more structured approach, with clear section headings and a logical flow of ideas. This would make it easier for readers to follow the arguments and key points.
2. The letter mentions concepts like 'helicopter research,' but it would be beneficial to provide more detailed explanations and examples to ensure that readers who may not be familiar with these terms can fully grasp their significance.

Does the article adequately reference differing views and opinions?

Yes

Are all factual statements correct, and are statements and arguments made adequately supported by citations?

Yes

Is the Open Letter written in accessible language?

Yes

Where applicable, are recommendations and next steps explained clearly for others to follow?

Partly

Is the rationale for the Open Letter provided in sufficient detail?

Yes

Reviewer Expertise:

Community engagement, infectious diseases, HIV/AIDS, Quantitative and qualitative data analysis

I confirm that I have read this submission and believe that I have an appropriate level of expertise to confirm that it is of an acceptable scientific standard, however I have significant reservations, as outlined above.

# Response

**Published:** 2023-11-22

**Authors:** Oluwafemi Adeagbo

**Affiliations:** Community and Behavioral Health, The University of Iowa, Iowa City, Iowa, USA

Dear Reviewer, Thank you very much for your insightful comments. We have have revised the manuscript as suggested. We have also checked the manuscript for logical flows. We highlighted some of the changes made in the revised manuscript. Thank you Oluwafemi Adeagbo PhD
